# Neurobiological Correlates of Inhibition of the Right Broca Homolog during New-Word Learning

**DOI:** 10.3389/fnhum.2016.00371

**Published:** 2016-07-28

**Authors:** Pierre Nicolo, Raphaël Fargier, Marina Laganaro, Adrian G. Guggisberg

**Affiliations:** ^1^Division of Neurorehabilitation, Department of Clinical Neurosciences, University Hospital GenevaGeneva, Switzerland; ^2^Laboratory of Cognitive Neurorehabilitation, Department of Clinical Neurosciences, Medical School, University of GenevaGeneva, Switzerland; ^3^Faculty of Psychology and Educational Sciences, University of GenevaGeneva, Switzerland

**Keywords:** rTMS, language, learning, picture naming, EEG

## Abstract

Repetitive transcranial magnetic stimulation (rTMS) has demonstrated beneficial effects on motor learning. It would be important to obtain a similar enhancement for verbal learning. However, previous studies have mostly assessed short-term effects of rTMS on language performance and the effect on learning is largely unknown. This study examined whether an inhibition of the right Broca homolog has long-term impact on neural processes underlying the acquisition of new words in healthy individuals. Sixteen young participants trained a new-word learning paradigm with rare, mostly unknown objects and their corresponding words immediately after continuous theta burst stimulation (cTBS) or sham stimulation of right inferior frontal gyrus (IFG) in a cross-over design. Neural effects were assessed with electroencephalography (EEG) source power analyses during the naming task as well as coherence analyses at rest 1 day before and after training. Inhibition of the right Broca homolog did not affect new word learning performance at the group level. Behavioral and neural responses to cTBS were variable across participants and were associated with the magnitude of resting-state alpha-band coherence between the stimulated area and the rest of the brain before stimulation. Only participants with high intrinsic alpha-band coherence between the stimulated area and the rest of the brain before stimulation showed the expected inhibition during naming and greater learning performance. In conclusion, our study confirms that cTBS can induce lasting modulations of neural processes which are associated with learning, but the effect depends on the individual network state.

## Introduction

Learning new words is an essential aspect of human language, which is not limited to childhood as adults also add new words to their mental lexicon. In addition, learning new words in adulthood is particularly relevant for the acquisition of a second language and during recovery in patients with aphasia.

The success of word learning in adults is often limited as compared to childhood, and in particular patients with aphasia often remain disabled despite high intensity speech therapy (Pedersen et al., 2004). Adjuvant interventions that enhance the outcome of learning and of speech and language therapy would therefore be highly welcome. One potential adjuvant therapy consists in neuromodulation via non-invasive cortical stimulation through repetitive transcranial magnetic stimulation (rTMS). RTMS can induce transient excitation or inhibition of cortical areas and their connected nodes beyond the duration of the stimulation (Najib et al., 2011). The rationale for inhibiting or exciting brain regions during word learning comes from the observation that successful acquisition of new words is associated with a dominant left hemispheric neural pattern before, during and after the training task (Breitenstein et al., 2005), while an overactivity of right language nodes may be deleterious (Hamilton et al., 2011). More importantly, good learners showed stronger activations of brain regions in the left hemisphere, in particular temporal regions, whereas poor learners activated preferentially a more diffuse bilateral network including right temporal and right inferior frontal areas (Wong et al., 2007; Mei et al., 2008). Similar observations were made in the context of word retrieval. Indeed, naming new words (as compared to familiar words) has been associated with increased activity in the left inferior frontal cortex (Broca’s area), the left temporal area and the left inferior parietal lobe (Cornelissen et al., 2004; Gronholm et al., 2005; Hulten et al., 2009). In post-stroke aphasia, a large body of research has suggested that the degree of activity in the left hemisphere was more critical for naming performance than activity in the right hemisphere (Thiel et al., 1998; Cao et al., 1999; Winhuisen et al., 2005).

On the other hand, some authors argue that the recruitment of the right hemisphere is not related to bad performance but rather to effortful language processing (Raboyeau et al., 2008) or to adaptation and plasticity after stroke (Blasi et al., 2002; Heiss et al., 2013). In consequence, the contribution of the right hemisphere to verbal learning is still under debate and the optimal parameters for rTMS are unknown.

There is some evidence that language skills may be facilitated by rTMS. Positive impact has been reported on speech repetition accuracy (Restle et al., 2012) or picture naming (Topper et al., 1998; Mottaghy et al., 1999, 2006). For instance, excitatory rTMS protocols over Wernicke’s area briefly decrease the response latency in a picture naming task in healthy people (Mottaghy et al., 2006). Inhibition of the right pars triangularis of the inferior frontal gyrus (IFG) led to decreased production latencies while inhibition of the right pars opercularis seemed to increase reaction times (Naeser et al., 2011). In stroke patients, several studies have revealed that inhibitory rTMS over the right Broca homolog can improve naming performance and/or decrease naming latencies in stroke patients or improve global language recovery (Martin et al., 2004; Naeser et al., 2011; Kindler et al., 2012; Thiel et al., 2013).

However, despite the evidence for momentary effects of rTMS on naming performance, it is largely unknown how it influences processes related to word learning. A better understanding of the long-term impact on word learning would be crucial for an application of rTMS as a complementary tool to speech therapy and language training. Such neurobiological information would likely lead to more efficient treatment methods in association with language therapies.

The present sham-controlled cross-over study therefore aimed to investigate the effect of rTMS on the ability to learn new words, as well as on the neural mechanisms related to naming new words in healthy participants. Unlike previous studies, we were not interested in short-term effects immediately after stimulation, but in long-term neural and behavioral effects on learning. Based on previous evidence for a negative influence of the right hemisphere and because of the excellent safety profile of inhibitory protocols which makes them easily applicable also in patients with recent brain lesions, we applied inhibitory rTMS over the right Broca homolog.

Neural changes were investigated with high-density electroencephalography (EEG). We first examined neural and behavioral effects at the group level in the hypothesis that rTMS modulates naming-related neural processing and neural interactions in language networks which translate into improved learning performance. Second, given that the behavioral effect of rTMS has been previously shown to be variable and dependent on the neural state of the brain before the stimulation (Silvanto and Pascual-Leone, 2008; Rizk et al., 2013; Nicolo et al., 2015; Vallence et al., 2015), we tested the hypothesis that network states before stimulation explain inter-individual variability in rTMS effects on learning and naming-related neural processing. In particular, alpha-band functional connectivity between the stimulated area and the rest of the brain was tested as a promising predictor of the response to rTMS (Rizk et al., 2013).

## Materials and Methods

### Subjects

Sixteen native French speakers (mean age 25 years, range 19–35, 4 men) without neurological or psychiatric disease were recruited after written informed consent. All were right-handed as determined by self-report and by the Edinburgh Handedness Scales (Oldfield, 1971) and had normal or corrected-to-normal visual acuity. All participants had comparable educational level (see Supplementary Table S1). Procedures were approved by the ethics committee of Geneva, Switzerland (project number: 10–220). All procedures were in accordance with international ethical standards on human experimentation and with the Helsinki Declaration of 1975, as revised in 2000. The participants were paid for their participation.

### Overview of Experimental Design

**FIGURE 1:**
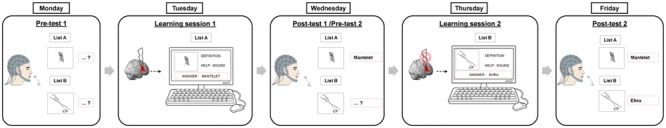
**Experimental design.** Participants (*n* = 16) underwent two learning sessions on Tuesdays and Thursdays. During each session, participants first received active or sham cTBS over the right Broca homolog followed by training of one list on a computer. One day before and after each training session, overt naming skills were tested for the learned and non-learned lists.

We performed a single-blinded within-subject sham-controlled crossover study. The complete procedure is summarized in **Figure 1** and took place over 5 days within the same week. Participants underwent two learning-to-name sessions on Tuesdays and Thursdays, during which they learned the name and the meaning of pictures of very rare objects. Immediately before learning, they received either inhibitory or sham stimulation over the right IFG, pars triangularis (Brodmann area 45) in counterbalanced order. Participants were blinded to the treatment arm. One day before and after each training session, i.e., on Mondays, Wednesdays, and Fridays, we obtained high-density EEG recording for pre- and post-tests of naming (event-related picture naming tasks) as well as during task-free resting states.

### Material

We used a picture naming task with rare real objects allowing the study of new word learning in healthy subjects with low probability of baseline knowledge while avoiding artificial pseudo-words. The stimuli consisted of 100 pictures of ancient or rare objects, tools or musical instruments and their corresponding words transformed in black and white line drawings (examples in Supplementary Material). All words have very low lexical frequency and were largely unknown at baseline. Two lists of 50 items (lists A and B) were constituted and matched on lexical frequency, first phoneme, phonological neighborhood and length in syllables and phonemes [from the French database Lexique (New et al., 2004), see details in Supplementary Table S2]. This allowed having a trained list and a control list in each learning session.

### Assessment of Naming Skills

Participants were tested individually in a soundproof dark room. The presentation of stimuli was controlled by E-Prime software (Psychology Software Tools, Inc., Sharpsburg, MD, USA). Pictures were presented in constant size of 240 pixels × 245 pixels (about 4.5° of visual angle) on a black screen (60 cm from their chest). In a familiarization phase, all 100 pictures and their corresponding names were presented on the screen one by one. Each item was presented for 3000 ms. In the picture naming task that followed, all items were presented in a pseudo-random order and were preceded by two warming-up filler trials (pictures of two familiar objects). An experimental trial began with a fixation cross presented for 500 ms. Then a blank screen preceded the appearance of the picture. Participants were requested to produce overtly the word corresponding to the picture as soon as they could. If they did not know the answer, they were asked to overtly say “no.” The picture remained on screen 3000 ms and a blank screen lasting 2000 ms was displayed before the next trial. Pictures of each list were presented twice (hence resulting in 100 trials per list) in pseudo-random order and in four separate blocks. The experiment lasted about 20 min with a break after each block.

*Learning performance* was computed as the difference in correct answers between post- and pre-training assessments.

Production latencies were measured by means of a voice key and were digitized for further systematic latency and accuracy check with speech analysis software (CheckVocal 2.2.6, Protopapas, 2007). Two kinds of incorrect responses were analyzed: no-responses (the participants indicated that he/she does not know the word by answering “no”) and errors (i.e., the participant produced a different name than the one expected for the picture, a phonologically transformed word and/or auto-corrections during articulation).

### Training Sessions

The learning task consisted of four runs of 50 pictures from lists A or B in pseudorandom order. The order of the two lists was counterbalanced between subjects. Pictures of the objects were presented in the black and white line drawing format and in a photograph picture format along with their definition on a computer screen. Participants were required to use the computer keyboard to write the corresponding word and then press ‘enter.’ If the word was unknown or incorrect, the individuals had the option of hearing the spoken word or reading the correct spelling of the word. Each picture stimulus remained on screen until the participant found or copied the correct picture-name association. Then, the next item was displayed automatically. The number of words produced correctly without help was computed after each run (four runs in total) and recorded as learning curve during training. For this learning stage, participants did not have time limits.

### Continuous Theta-Burst Stimulation (cTBS)

Continuous theta burst stimulation (cTBS), a more recent form of rTMS, has been shown to induce a decrease of neural activity in a specific network (Nyffeler et al., 2006). TBS has the advantage of inducing longer aftereffects while requiring shorter stimulation time than conventional rTMS (Huang et al., 2005; Nyffeler et al., 2006).

CTBS was delivered with a biphasic waveform through a MagPro X100 system (Medtronic Functional Diagnostics). The stimulator was connected with a figure-of-eight coil (MCF-B65) with a diameter of 2 mm × 75 mm and a geometrically identical coil for the sham condition (MCF-P-B65). Sham stimulation produced the same noise as true stimulation, but induced no magnetic field within the underlying neural tissue.

The cTBS protocol consisted of continuous trains of 801 pulses, applied in 267 bursts. Each burst contained three pulses at 30 Hz with an interburst interval of 167 ms. Total duration of a train was 44 s (Kindler et al., 2012).

The coil was positioned over the right posterior inferior frontal gyrus (pIFG, pars triangularis, BA 45) using landmarks of the international 10–20 EEG system. The Münster T2T-Converter (Deppe et al., 2003) coordinates were used to calculate the scalp position relative to EEG coordinates which corresponded to this specific localization. Stimulation was applied between electrodes C4 and F8 (10–20, 1.26/0.74; Talairach space x/yr/z, 58/31/22). The coil was held tangentially to the skull with the handle pointing upward. Stimulation intensity was expressed as percentage of stimulator output and was set to 90% of the individual resting motor threshold of the small left hand muscles (Kindler et al., 2012).

Adverse events were checked after each rTMS intervention and on subsequent days by the investigators including an experimented neurologist. No negative side effects were observed except for transient discomfort during stimulation.

### EEG Recordings and Preprocessing

Electroencephalography was recorded during a task-free resting state and during the picture naming tasks (event related) at each pre-learning and post-learning session. An Active-Two EEG system (Biosemi V.O.F., Amsterdam, The Netherlands) with 128 electrodes was used to digitize signals at a rate of 512 Hz (filters: DC to 104 Hz, 3 dB/octave slope).

Resting-state recordings were obtained continuously for 10 min while participants were awake and kept their eyes-closed. For event-related recordings during naming, epochs lasting from 600 ms before to 1000 ms after the picture onset were extracted. Subjects were instructed to avoid eye movements and blinking, swallowing or any movement other than required for the task.

Epochs or resting-state periods with movement artifacts, eye blinking, other noise, or signs of sleepiness were excluded by off-line visual inspection. Bad channels containing prolonged artifacts were ignored from further analyses. Artifact-free epochs were recalculated against the average reference (all non-excluded channels).

### Source Reconstruction

Analyses in source space were performed using the software *Matlab* (The MathWorks Inc.) with the open-source toolbox NUTMEG^1^ (Dalal et al., 2011) and its functional connectivity mapping (FCM) toolbox (Guggisberg et al., 2011). A lead-potential with 10 mm grid spacing (894 voxels) was computed using a spherical head model with anatomical constraints (Spinelli et al., 2000) based on the segmented gray matter of the standard Montreal Neurological Institute (MNI) brain. An adaptive spatial filter (scalar minimum variance beamformer) was used to reconstruct neural oscillations in source space from surface sensors (Sekihara et al., 2004).

### Time-Frequency Decomposition

Event-related power changes (erPOW; Pfurtscheller and Andrew, 1999) were assessed to investigate oscillatory modulation of sets of neurons in response to the naming task.

Adaptive spatial filter weights were calculated for each subject from all artifact-free epochs of a given condition. In order to optimize the beamformer for the respective frequency bands, we obtained separate weights for each of the following bands: 1–20, 21–30, 31–45, and 55–95 Hz (Dalal et al., 2008).

Signals were Fourier-transformed using a sliding Hanning window of 500 ms width shifted in time steps of 50 ms. Fourier coefficients were projected to source space with the adaptive spatial filter and power was computed at each time window across all trials for each of the following frequency bands: delta (2–3 Hz), theta (4–7 Hz), alpha (8–12 Hz), low beta (13–20 Hz), high beta (21–30 Hz), gamma (31–45 Hz), and high-gamma (55–95 Hz). Time-frequency power values were log transformed and baseline corrected by subtracting a pre-stimulus baseline power average from -600 to 0 ms.

### Resting-State Coherence (rsCOH)

Adaptive spatial filter weights were calculated for each subject from 300 artifact-free resting-state epochs of 1 s duration in the bandwidth from 1 to 20 Hz.

Functional connectivity between two time series was quantified as the imaginary component of coherence (IC) (Nolte et al., 2004). Although conservative, this index was chosen because it is robust to volume conduction and avoids distortions due to spatial leakage of inverse solutions (Sekihara et al., 2011).

Imaginary component of coherence was computed in source space across 5 min of artifact-free epochs for four standard frequency bands: delta (1–3 Hz), theta (4–7 Hz), alpha (8–12 Hz), and low beta (13–20 Hz). In order to obtain a global measure of functional connectivity at each voxel, we computed the graph theoretical measure of node degree in weighted networks (Newman, 2004) as the summed IC with all other voxels. This measure indicates the overall importance of each voxel in the brain network (Stam and van Straaten, 2012).

Variations in functional connectivity magnitude can be due to fluctuations in signal to noise ratio between participants or conditions. To avoid this problem, node degree maps were normalized. This was achieved by subtracting the mean node degree of all voxels of the subject from the values at each individual voxel and by dividing by the standard deviation over all voxels, hence obtaining *z*-scores (Guggisberg et al., 2015).

### Statistical Analyses

For behavioral measures, repeated measures ANOVAs were computed on rates of correct productions with session (pre-learning, post-learning) and stimulation condition (true, sham) as within subject factors.

The brain region that we targeted with cTBS, i.e., the right pIFG (Brodmann area 45), as well as its left homolog, were defined as regions of interest (ROIs) using the automated anatomical labeling (AAL) atlas (Tzourio-Mazoyer et al., 2002). An additional ROI was functionally defined as the area showing significant EEG activation during picture naming at pre-test 1 (*p* < 0.05, 5% false discovery rate, FDR).

Event-related power changes and resting-state coherence (rsCOH) at each ROI were tested against the null hypotheses of zero mean with a *t*-test for one sample, as well as against the null hypothesis of zero difference between before and after learning, or between true and sham stimulation, with paired *t*-tests. Furthermore, we tested neural changes for correlations with learning effects using a Pearson correlation analysis. Normal distribution of variables was confirmed by visual inspection of the data as well as with a Kolmogorov-Smirnov goodness-of-fit test.

To correct for the family wise error of testing ROI event-related power at multiple time-frequency data points, we performed a permutation test. In brief, at each of 2000 permutation loops, the values of a random combination of subjects were inverted by multiplication with -1 and subjected to the same statistical test. The largest number of significant consecutive windows at *p* < 0.05 (uncorrected) across all data points was recorded. The significance of the number of consecutive windows in the real dataset was then determined from its position in the empirical distribution obtained through permutations. Since we compare against the maximum value across all data points, we effectively control for multiple testing. Time-frequency windows that belonged to a window cluster that was larger than 95% of clusters obtained in permutations were considered significant at *p* < 0.05, corrected.

## Results

### Behavioral Results

**FIGURE 2:**
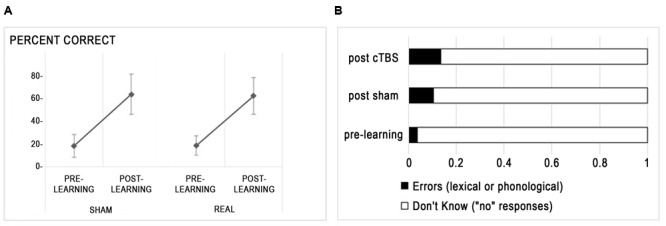
**Behavioral effects of rTMS. (A)** Percent correct responses (mean ± standard deviation) are displayed for each stimulation condition. **(B)** Distribution of incorrect responses before and after learning.

At pre-test, naming scores did not differ between A and B lists (*t* < 1). **Figure 2A** presents the improvement of naming scores from pre-learning to post-learning sessions in each stimulation condition. There was a main effect of session indicating improved naming after training [*F*(1,15) = 195.3, *p* < 0.0001], no main effect of stimulation type (*F* < 1) and no interaction between session and stimulation (*F* < 1). The between subject variability in learning was high, with learnt words varying from 13 to 70%. Regarding naming errors, whereas the amount of responses indicating that the participant did not know the word (“no” responses) were bound to decrease after training, other kinds of erroneous responses (i.e., lexical errors, mainly confusion between learnt words, and phonological errors) increased (see **Figure 2B**), with a trend for increased rates of errors after cTBS compared to sham [*t*(15) = -2.11, *p* = 0.052].

**FIGURE 3:**
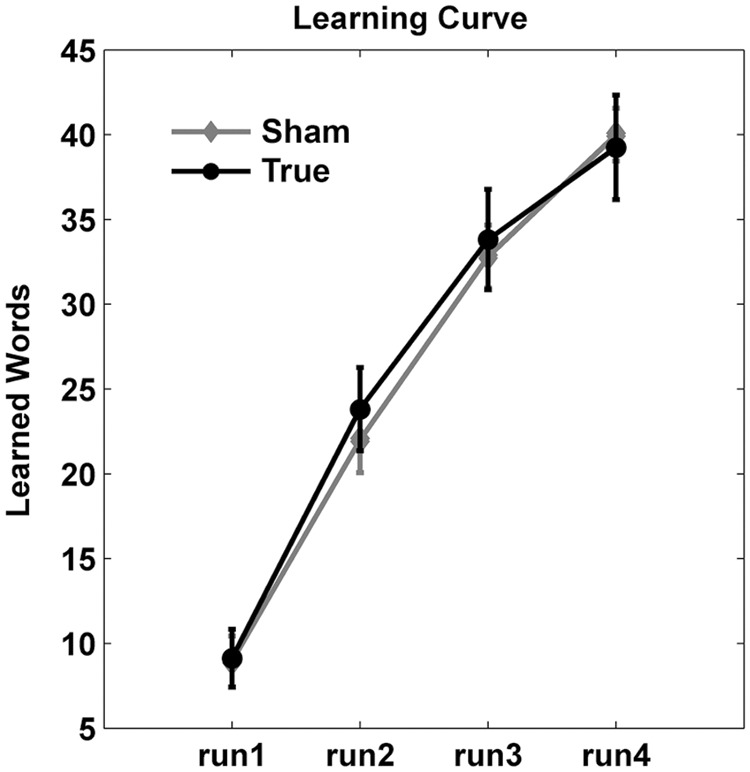
**Learning curves.** Number of correct responses (mean ± standard deviation) are shown across the four training runs. Overall, cTBS did not enhance training gains.

**Figure 3** shows the training curve over the four runs during the training sessions. Accuracy improved over the four training runs as evidenced by a main effect of run [*F*(3,115) = 108.4, *p* < 0.0001]. However, there was no significant main effect of stimulation type (*F* < 0.2) nor an interaction between run and stimulation (*F* < 0.7).

Hence, inhibition of the right Broca homolog with cTBS did not influence the ability to learn new words at the group level but slightly increased the amount of errors relative to “don’t know” responses.

### EEG Results

**FIGURE 4:**
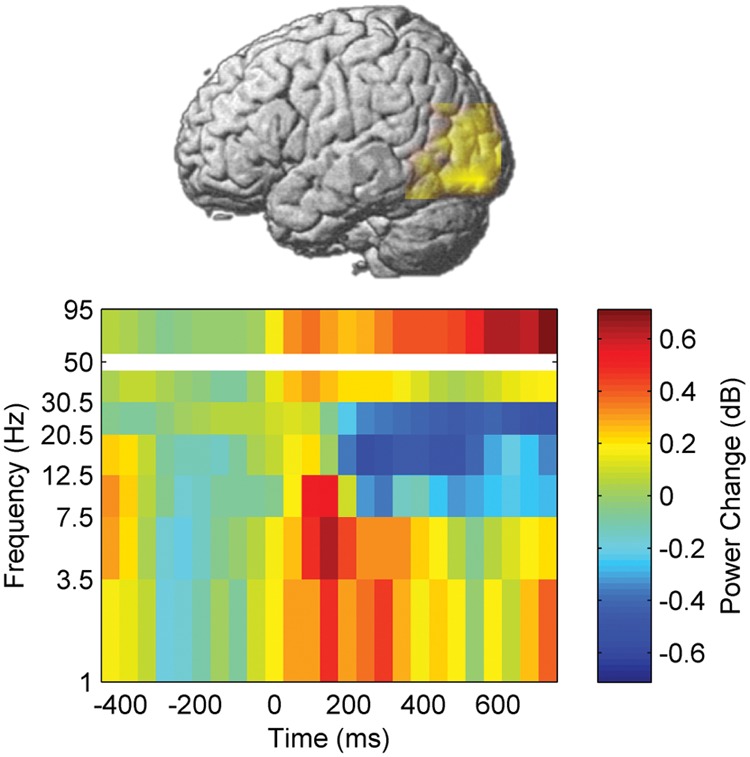
**Naming-related power changes before training.** Time 0 corresponds to picture presentation.

We first investigated whether the neural processing of word naming was modulated by learning and stimulation by assessing event-related power changes. A voxel-wise time-frequency decomposition during the pre-training session (pre-test 1) revealed a significant cluster of naming-related power modulation which survived correction for testing multiple voxels, time windows, and frequency bands (*p* < 0.05, FDR corrected). High-gamma (50–95 Hz) power increased between 25 and 800 ms after picture presentation in a left temporo-occipital brain area (**Figure 4**, peak MNI coordinates: -42 -90 20). This area was therefore defined as ROI for neural processing of naming.

**FIGURE 5:**
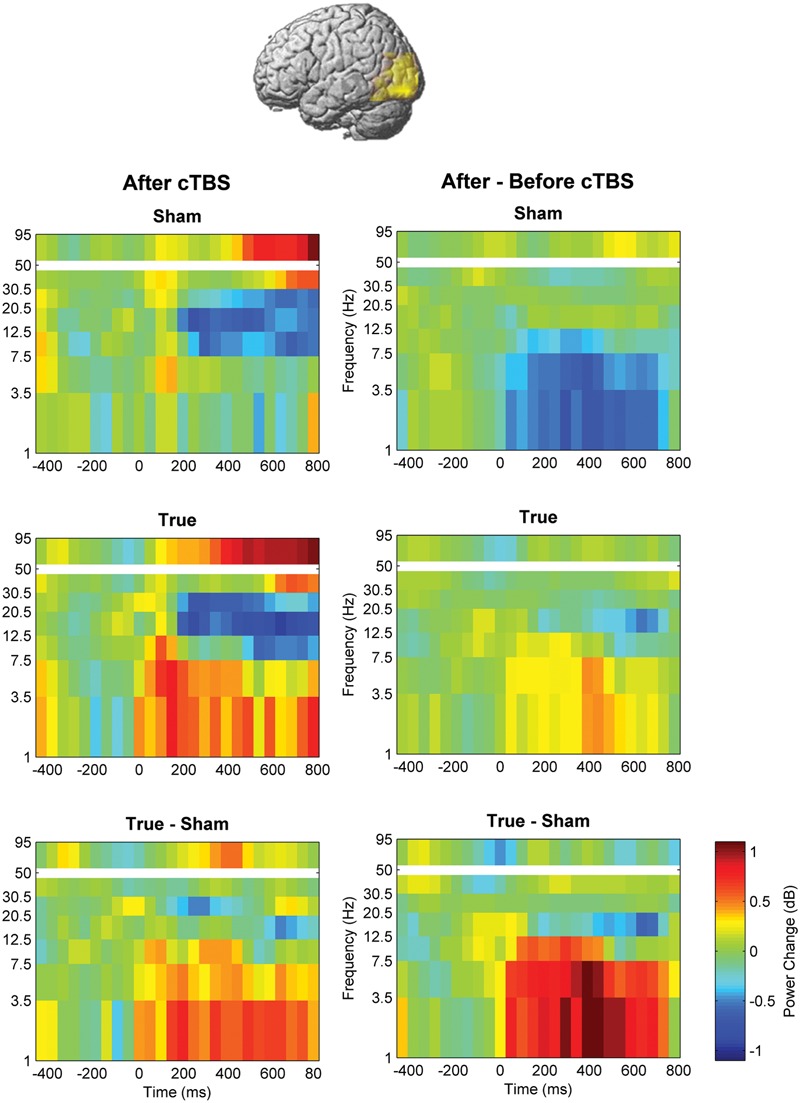
**Comparison of induced brain responses over the left temporo-occipital region.** The panels in the left column show naming-related power changes 1 day after stimulation, whereas the right-hand panels represent the difference (after minus before). Significant power increase at low frequencies was observed after cTBS in comparison to sham.

**FIGURE 6:**
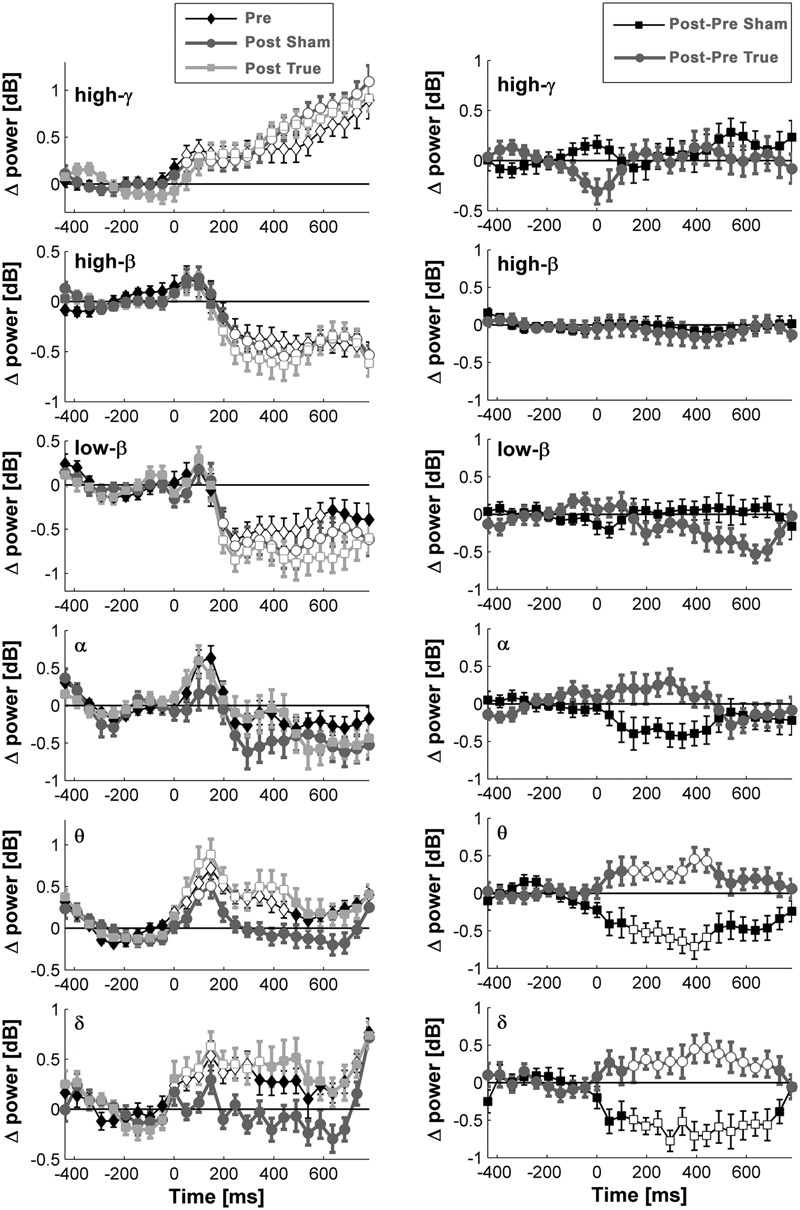
**Naming- and stimulation-related power modulations at the left temporo-occipital ROI.** The left column shows power modulations (mean ± standard error of mean) before (pre) and after each type of stimulation (post), relative to baseline. The right column visualizes changes induced by stimulation. Empty symbols represent significant time windows (*p* < 0.05, corrected). Theta and delta power increased 1 day after true cTBS, whereas the opposite was observed after sham stimulation.

Besides the increase in high-gamma power, naming during the pre-training session also induced, in the left temporo-occipital ROI, a power decrease in beta frequency bands between 200 and 800 ms after stimulus presentation. In contrast, increased power was observed in theta band between 0 and 465 ms as well as in delta band between 0 and 320 ms (*p* < 0.05, corrected, **Figures 4–6**).

Beta and high-gamma power modulations remained unchanged at the post-test 1 day after training (**Figure 5**). In contrast, delta and theta band power modulations were significantly altered 1 day after learning and the alteration depended significantly on the stimulation condition. Theta and delta power modulations decreased 1 day after learning preceded by sham stimulation (*p* < 0.05, corrected), whereas the opposite occurred after true stimulation (*p* < 0.05, corrected). The difference between true and sham condition was significant (*p* < 0.05, corrected). This effect was observed during a time window between 120 and 460 ms after stimulus presentation for the theta band and between 120 and 710 ms for the delta band. However, theta and delta band power changes were not associated with learning performance (max correlation at any time point *r* < 0.49, *p* > 0.05 uncorrected) or with naming errors (*r* < 0.45, *p* > 0.05, uncorrected) in any stimulation condition.

No learning and stimulation effects were found at the left and right inferior frontal ROIs or on rsCOH 1 day after learning at any ROI.

As stated in the Introduction, the effect of rTMS can depend on neural states before the stimulation. To test for state-dependency in our study, we examined the relationship between resting-state connectivity in the alpha frequency band, event-related neural changes and behavioral effects induced by cTBS over the right IFG.

**FIGURE 7:**
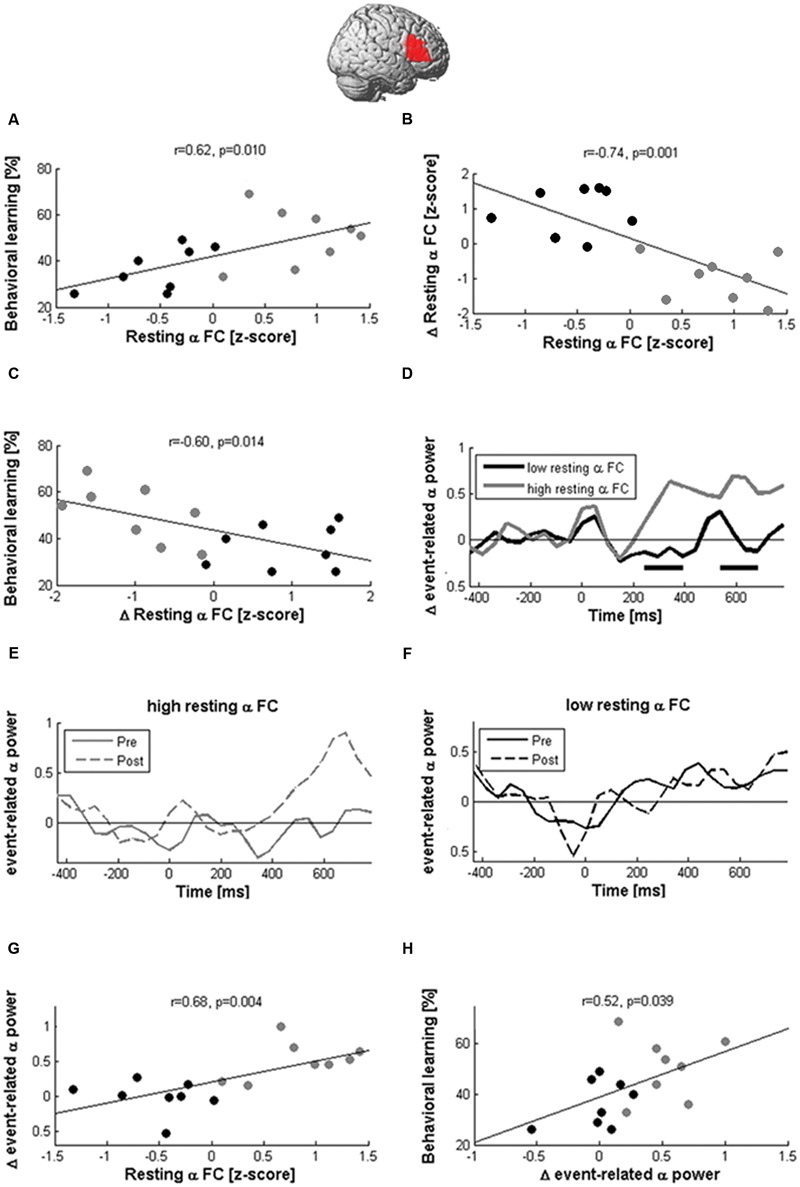
**Network states modulate the effects of cTBS.** Learning and EEG after-effects of cTBS depended on the magnitude of resting-state alpha coherence between the right IFG and the rest of the brain before stimulation. Participants with large pre-stimulation coherence learned more words **(A)** and decreased alpha-band coherence after stimulation **(B)**, while participants with low pre-stimulation connectivity showed the opposite pattern. The reduction of coherence between the right Broca homolog and the rest of the brain was correlated with better learning performance **(C)**. In participants with high pre-stimulation coherence, cTBS also produced the expected local inhibition during naming, as indicated by an increase of event-related alpha power (**D,E**; black rectangles indicate significant differences at *p* < 0.05). In contrast, participants with low pre-stimulation alpha-band coherence showed no power modulation after stimulation **(F)**. Hence, resting-state coherence (rsCOH) before stimulation was associated with greater alpha power increases between 200 and 800 ms after picture presentation **(G)**, which was in turn correlated with better learning **(H)**.

A significant positive correlation was observed between resting-state connectivity in the alpha band before stimulation and learning performance (*r* = 0.62, *p* = 0.01; **Figure 7A**) indicating larger new-word learning in participants with greater alpha-band connectivity in the right IFG. This was the case only for true stimulation and not observed in the sham condition (*r* = 0.23, *p* = 0.37).

Although cTBS had no significant effect on rsCOH at the group level, it decreased alpha-band resting-state connectivity in participants with high pre-stimulation connectivity but increased values in participants with low pre-stimulation connectivity as indicated by the high negative correlation (*r* = -0.74, *p* = 0.001; **Figure 7B**) between pre-stimulation and change in resting-state connectivity in the alpha band. Furthermore, connectivity changes at the alpha-band induced by stimulation were negatively correlated with learning performance (*r* = -0.6, *p* = 0.014; **Figure 7C**) and positively correlated with naming errors after stimulation (*r* = 0.51, *p* = 0.041). This indicated better new-word learning performance and lower lexical confusions in participants in whom resting-state connectivity between the right Broca homolog and the rest of the brain was decreased by stimulation.

Alpha-band coherence before stimulation further influenced naming-related alpha power modulations (**Figures 7D–F**). In participants with high resting-state alpha-band coherence before stimulation, cTBS produced an enhancement of naming-related alpha power at the right Broca homolog, while it produced no change or even decreased power in participants with low baseline coherence. We then averaged the naming-related alpha power changes of each participant across all time windows >200 ms after picture presentation and found a positive correlation with baseline alpha-band coherence (*r* = 0.68, *p* = 0.004; **Figure 7G**). In addition, a significant positive correlation was also found between event-related alpha power increases and learning performance (*r* = 0.52, *p* = 0.039; **Figure 7H**).

These pattern of correlations in the alpha band were specific to cTBS stimulation and not observed after sham condition (*r* < 0.32, *p* > 0.05). Furthermore, they were not observed in other frequency bands (max correlations with changes at right IFG coherence for beta, theta, delta bands *r* < 0.430, *p* > 0.05).

When gender was included as confounding factor, all correlations remained significant (*p* < 0.03) and unpaired *t*-tests (men vs. women) on our variables were not significant (*p* > 0.3). The associations were therefore not driven by gender differences.

## Discussion

Non-invasive brain stimulation can enhance learning through practice in healthy people or during recovery of neurological deficits (Tanaka et al., 2011; Wessel et al., 2016). This has been shown in particular for motor learning (Reis et al., 2008; Herzfeld et al., 2014). The present study examined if a similar benefit can be obtained for verbal learning, and, if yes, by which underlying neural mechanisms.

Overall, a single session of inhibition of the right Broca homolog did not facilitate new word learning nor impact the learning curve during the training phase. Our concomitant monitoring of neural effects with EEG provides insights into the reasons for this lack of benefit. Although our protocol did induce long-term modulations of naming-related oscillations in left temporo-occipital language areas, they were not sufficient for improving behavioral learning. The lack of benefit at the group level seemed to be due to endogenous variability of the neural state at baseline. The neural and behavioral response to cTBS in an individual depended on the state of intrinsic functional connectivity of the stimulated brain region. Participants with high intrinsic alpha-band coherence between the target area and the rest of the brain before stimulation were more likely to show the expected inhibition and showed greater learning performance. Participants with low intrinsic alpha-band coherence between right inferior frontal regions and the rest of the brain before stimulation showed a paradoxical increase in coherence after cTBS and showed poorer verbal learning.

### Neural Processing during Naming and Its Modulation after cTBS

Time-frequency analyses revealed power modulations of oscillations in several frequency bands prior and after training. Increased power in the high-gamma band and decreased power in beta bands in the left temporo-occipital region were not modulated as a function of learning and therefore likely reflect invariant neural processes during word naming. The activation of the temporo-occipital junction, the modulations in gamma and beta bands and their time course suggest that these neural modulations correspond to visual object processing (Bookheimer et al., 1998). Beta power suppression can reflect working memory in naming (Piai et al., 2015). However, its time line (the first 800 msec post picture onset) and the combination with gamma rhythm in occipital regions rather support functional mechanisms of visual processing (Michalareas et al., 2016) and (visual) working memory (Honkanen et al., 2015). Honkanen et al. (2015) recently showed that gamma oscillations in the visual cortex are involved in the extraction and maintenance of object features and associated to the content of visual working memory. These results are also compatible with the observation that the occipital cortex remains active during a large part of the naming processes (Hassan et al., 2015).

Delta and theta oscillations were also modulated during naming and were further influenced by learning and stimulation. Theta oscillations, in particular, have been previously described in association with working memory load (Fuentemilla et al., 2010) or encoding and retrieval of episodic memory (Hasselmo and Stern, 2014). The modulations observed in the theta band in the first 500 ms post picture onset are in line with lexical processes occurring in this time-window (Indefrey, 2011) and are compatible with recent report that theta oscillations are affected by lexical frequency (Piai et al., 2014) although in a different time-window. In the present study, the difference between pre- and post- training corresponds to the difference between unknown and familiar words, which may mimic the lexical frequency effect. Delta oscillations have been associated with a large variety of cognitive processes including motivational drive (Knyazev, 2012), inhibition of sensorial afferences or attention to internal processing during mental tasks (Harmony, 2013), memory retrieval (Fernandez et al., 1998), and cortical plasticity (Assenza and Di Lazzaro, 2015; Assenza et al., 2015). However, theta-delta modulations induced by inhibition of the right Broca homolog were not sufficient for improving language learning, hence suggesting that they do not reflect critical processes for new-word learning.

Since we observed a naming-related processing mainly in the left temporo-occipital junction, it might be more efficient to target this area directly. A recent study using anodal (excitatory) tDCS over Wernicke’s area, which is located much more closely to the left temporo-occipital junction, has indeed reported a facilitation of new word acquisition in healthy people (Meinzer et al., 2014).

It might be tempting to speculate that more than a single stimulation session would have been necessary to obtain learning effects at the group level. However, since we do observe robust neural effects even after one session, we consider this possibility unlikely, although we cannot exclude that more sessions may have reduced inter-individual variability.

### State-Dependency and Variability in Response to cTBS

The main reason for the lack of an overall behavioral effect at the group level is the large inter-individual variability in the response to cTBS. Our EEG monitoring identified one particular factor contributing to this variability. Behavioral and neural effects of cTBS were dependent on pre-stimulation alpha-band coherence between the stimulated area and the rest of the brain. Such a dependency of the response to stimulation on the neural state before stimulation has been well known for local neural activations at the stimulation site (Silvanto and Pascual-Leone, 2008; Silvanto et al., 2008). Alpha-band coherence was also previously reported to shape the behavioral effect of cTBS at the right parietal cortex on spatial attention (Rizk et al., 2013). Here, we reproduced a state-dependency at the network level in an independent population, at a different network, and for a different behavior, hence suggesting that this represents a general response pattern of the human brain to cTBS. Moreover, we identified mechanisms by which rsCOH may influence the response to stimulation. Alpha-band coherence was correlated with increases in event-related alpha power after stimulation, hence suggesting that resting-state network interactions translate into specific local patterns of brain oscillations during naming. Event-related alpha power increases are thought to represent inhibition during tasks in order to liberate neural resources at more critical brain areas (Pfurtscheller, 1992; Klimesch et al., 2007; Jensen et al., 2012). Hence, it seems that, in participants with large pre-stimulation alpha-band coherence between the stimulated area and the rest of the brain, our stimulation protocol had the intended effect of local inhibition during learning and naming and, consequently, improved learning. However, no or even the opposite effect seems to have occurred in about half of the participants who had relatively low baseline coherence. This may suggest that cTBS did not have a strictly inhibiting effect in all participants, but may even have produced a paradoxical excitation in some cases.

Other factors may also have contributed to variability in the response to stimulation, such as, e.g., genetics (Cheeran et al., 2008), cortical physiology (Hamada et al., 2013), and hormones (Clow et al., 2014).

### Local Effects of cTBS

In comparison to previous studies on EEG effects of rTMS (Fuggetta et al., 2008; Hamidi et al., 2009; Veniero et al., 2011; Noh et al., 2012; Vernet et al., 2013), we did not observe robust effects on rsCOH or erPOW under the stimulation site at the group level. This is explained by the fact that our recordings were made 1 day after stimulation, when excitability modulations are expected to have disappeared (Grossheinrich et al., 2009; Noh et al., 2012; Rizk et al., 2013; Vernet et al., 2013). Yet, our findings suggest that the neural response to stimuli can remain modified beyond the duration of traditional aftereffects.

## Conclusion

In order to improve the efficacy of rTMS as adjuvant language therapy in the future, it will be critical to understand the neural signatures of verbal learning that need to be modulated in learning. Furthermore, the present results indicate that there is a need to understand and control factors underlying inter-individual variability in order to apply stimulation more selectively in participants who are likely to respond. Thereby, our results support the usefulness of EEG network imaging for monitoring and predicting the aftereffects induced by non-invasive brain stimulation.

## Author Contributions

PN acquired data, performed EEG analyses, and wrote the paper. RF acquired data, performed behavioral analyses, and wrote the paper. ML designed the study, acquired data, performed behavioral analyses, and wrote the paper. AG designed the study, acquired data, performed EEG analyses, and wrote the paper.

## Conflict of Interest Statement

The authors declare that the research was conducted in the absence of any commercial or financial relationships that could be construed as a potential conflict of interest.

## Footnotes

1 http://nutmeg.berkeley.edu

## References

[B1] Assenza G. Di Lazzaro V. (2015). A useful electroencephalography (EEG) marker of brain plasticity: delta waves. *Neural Regen. Res.* 10 1216–1217. 10.4103/1673-5374.162698 26487841 PMC4590226

[B2] Assenza G. Pellegrino G. Tombini M. Di Pino G. Di Lazzaro V. (2015). Wakefulness delta waves increase after cortical plasticity induction. *Clin. Neurophysiol.* 126 1221–1227. 10.1016/j.clinph.2014.09.029 25631611

[B3] Blasi V. Young A. C. Tansy A. P. Petersen S. E. Snyder A. Z. Corbetta M. (2002). Word retrieval learning modulates right frontal cortex in patients with left frontal damage. *Neuron* 36 159–170. 10.1016/S0896-6273(02)00936-4 12367514

[B4] Bookheimer S. Y. Zeffiro T. A. Blaxton T. A. Gaillard W. D. Malow B. Theodore W. H. (1998). Regional cerebral blood flow during auditory responsive naming: evidence for cross-modality neural activation. *Neuroreport* 9 2409–2413. 10.1097/00001756-199807130-00047 9694237

[B5] Breitenstein C. Jansen A. Deppe M. Foerster A. F. Sommer J. Wolbers T. (2005). Hippocampus activity differentiates good from poor learners of a novel lexicon. *Neuroimage* 25 958–968. 10.1016/j.neuroimage.2004.12.019 15808996

[B6] Cao Y. Vikingstad E. M. George K. P. Johnson A. F. Welch K. M. (1999). Cortical language activation in stroke patients recovering from aphasia with functional MRI. *Stroke* 30 2331–2340. 10.1161/01.STR.30.11.2331 10548667

[B7] Cheeran B. Talelli P. Mori F. Koch G. Suppa A. Edwards M. (2008). A common polymorphism in the brain-derived neurotrophic factor gene (BDNF) modulates human cortical plasticity and the response to rTMS. *J. Physiol.* 586 5717–5725. 10.1113/jphysiol.2008.159905 18845611 PMC2655403

[B8] Clow A. Law R. Evans P. Vallence A. M. Hodyl N. A. Goldsworthy M. R. (2014). Day differences in the cortisol awakening response predict day differences in synaptic plasticity in the brain. *Stress* 17 219–223. 10.3109/10253890.2014.905533 24646342

[B9] Cornelissen K. Laine M. Renvall K. Saarinen T. Martin N. Salmelin R. (2004). Learning new names for new objects: cortical effects as measured by magnetoencephalography. *Brain Lang.* 89 617–622. 10.1016/j.bandl.2003.12.007 15120553

[B10] Dalal S. S. Guggisberg A. G. Edwards E. Sekihara K. Findlay A. M. Canolty R. T. (2008). Five-dimensional neuroimaging: localization of the time-frequency dynamics of cortical activity. *Neuroimage* 40 1686–1700. 10.1016/j.neuroimage.2008.01.023 18356081 PMC2426929

[B11] Dalal S. S. Zumer J. M. Guggisberg A. G. Trumpis M. Wong D. D. Sekihara K. (2011). MEG/EEG source reconstruction, statistical evaluation, and visualization with NUTMEG. *Comput. Intell. Neurosci.* 2011 758973 10.1155/2011/758973 PMC3061455 21437174

[B12] Deppe M. Steinsträter O. Sommer J. Besmens V. Knecht S. (2003). The T2T-database java applet. *Neuroimage* 19 S48.

[B13] Fernandez G. Weyerts H. Tendolkar I. Smid H. G. Scholz M. Heinze H. J. (1998). Event-related potentials of verbal encoding into episodic memory: dissociation between the effects of subsequent memory performance and distinctiveness. *Psychophysiology* 35 709–720. 10.1111/1469-8986.3560709 9844432

[B14] Fuentemilla L. Penny W. D. Cashdollar N. Bunzeck N. Duzel E. (2010). Theta-coupled periodic replay in working memory. *Curr. Biol.* 20 606–612. 10.1016/j.cub.2010.01.057 20303266 PMC2856918

[B15] Fuggetta G. Pavone E. F. Fiaschi A. Manganotti P. (2008). Acute modulation of cortical oscillatory activities during short trains of high-frequency repetitive transcranial magnetic stimulation of the human motor cortex: a combined EEG and TMS study. *Hum. Brain Mapp.* 29 1–13. 10.1002/hbm.20371 17318833 PMC6870897

[B16] Gronholm P. Rinne J. O. Vorobyev V. Laine M. (2005). Naming of newly learned objects: a PET activation study. *Brain Res. Cogn. Brain Res.* 25 359–371. 10.1016/j.cogbrainres.2005.06.010 16095887

[B17] Grossheinrich N. Rau A. Pogarell O. Hennig-Fast K. Reinl M. Karch S. (2009). Theta burst stimulation of the prefrontal cortex: safety and impact on cognition, mood, and resting electroencephalogram. *Biol. Psychiatry* 65 778–784. 10.1016/j.biopsych.2008.10.029 19070834

[B18] Guggisberg A. G. Dalal S. S. Zumer J. M. Wong D. D. Dubovik S. Michel C. M. (2011). Localization of cortico-peripheral coherence with electroencephalography. *Neuroimage* 57 1348–1357. 10.1016/j.neuroimage.2011.05.076 21672634

[B19] Guggisberg A. G. Rizk S. Ptak R. Di Pietro M. Saj A. Lazeyras F. (2015). Two intrinsic coupling types for resting-state integration in the human brain. *Brain Topogr.* 28 318–329. 10.1007/s10548-014-0394-2 25182143

[B20] Hamada M. Murase N. Hasan A. Balaratnam M. Rothwell J. C. (2013). The role of interneuron networks in driving human motor cortical plasticity. *Cereb. Cortex* 23 1593–1605. 10.1093/cercor/bhs147 22661405

[B21] Hamidi M. Slagter H. A. Tononi G. Postle B. R. (2009). Repetitive transcranial magnetic stimulation affects behavior by biasing endogenous cortical oscillations. *Front. Integr. Neurosci.* 3:14 10.3389/neuro.07.014.2009 PMC2707056 19587850

[B22] Hamilton R. H. Chrysikou E. G. Coslett B. (2011). Mechanisms of aphasia recovery after stroke and the role of noninvasive brain stimulation. *Brain Lang.* 118 40–50. 10.1016/j.bandl.2011.02.005 21459427 PMC3109088

[B23] Harmony T. (2013). The functional significance of delta oscillations in cognitive processing. *Front. Integr. Neurosci.* 7:83 10.3389/fnint.2013.00083 PMC3851789 24367301

[B24] Hassan M. Benquet P. Biraben A. Berrou C. Dufor O. Wendling F. (2015). Dynamic reorganization of functional brain networks during picture naming. *Cortex* 73 276–288. 10.1016/j.cortex.2015.08.019 26478964

[B25] Hasselmo M. E. Stern C. E. (2014). Theta rhythm and the encoding and retrieval of space and time. *Neuroimage* 85(Pt 2) 656–666. 10.1016/j.neuroimage.2013.06.022 23774394 PMC3918488

[B26] Heiss W. D. Hartmann A. Rubi-Fessen I. Anglade C. Kracht L. Kessler J. (2013). Noninvasive brain stimulation for treatment of right- and left-handed poststroke aphasics. *Cerebrovasc. Dis.* 36 363–372. 10.1159/000355499 24217362

[B27] Herzfeld D. J. Pastor D. Haith A. M. Rossetti Y. Shadmehr R. O’shea J. (2014). Contributions of the cerebellum and the motor cortex to acquisition and retention of motor memories. *Neuroimage* 98 147–158. 10.1016/j.neuroimage.2014.04.076 24816533 PMC4099269

[B28] Honkanen R. Rouhinen S. Wang S. H. Palva J. M. Palva S. (2015). Gamma oscillations underlie the maintenance of feature-specific information and the contents of visual working memory. *Cereb. Cortex* 25 3788–3801. 10.1093/cercor/bhu263 25405942

[B29] Huang Y. Z. Edwards M. J. Rounis E. Bhatia K. P. Rothwell J. C. (2005). Theta burst stimulation of the human motor cortex. *Neuron* 45 201–206. 10.1016/j.neuron.2004.12.033 15664172

[B30] Hulten A. Vihla M. Laine M. Salmelin R. (2009). Accessing newly learned names and meanings in the native language. *Hum. Brain Mapp.* 30 976–989. 10.1002/hbm.20561 18412130 PMC6870721

[B31] Indefrey P. (2011). The spatial and temporal signatures of word production components: a critical update. *Front. Psychol.* 2:255 10.3389/fpsyg.2011.00255 PMC3191502 22016740

[B32] Jensen V. F. Emborg H. D. Aarestrup F. M. (2012). Indications and patterns of therapeutic use of antimicrobial agents in the Danish pig production from 2002 to 2008. *J. Vet. Pharmacol. Ther.* 35 33–46. 10.1111/j.1365-2885.2011.01291.x 21564137

[B33] Kindler J. Schumacher R. Cazzoli D. Gutbrod K. Koenig M. Nyffeler T. (2012). Theta burst stimulation over the right Broca’s homologue induces improvement of naming in aphasic patients. *Stroke* 43 2175–2179. 10.1161/STROKEAHA.111.647503 22581821

[B34] Klimesch W. Sauseng P. Hanslmayr S. (2007). EEG alpha oscillations: the inhibition-timing hypothesis. *Brain Res. Rev.* 53 63–88. 10.1016/j.brainresrev.2006.06.003 16887192

[B35] Knyazev G. G. (2012). EEG delta oscillations as a correlate of basic homeostatic and motivational processes. *Neurosci. Biobehav. Rev.* 36 677–695. 10.1016/j.neubiorev.2011.10.002 22020231

[B36] Martin P. I. Naeser M. A. Theoret H. Tormos J. M. Nicholas M. Kurland J. (2004). Transcranial magnetic stimulation as a complementary treatment for aphasia. *Semin. Speech Lang.* 25 181–191. 10.1055/s-2004-825654 15118944

[B37] Mei L. Chen C. Xue G. He Q. Li T. Xue F. (2008). Neural predictors of auditory word learning. *Neuroreport* 19 215–219. 10.1097/WNR.0b013e3282f46ea9 18185111

[B38] Meinzer M. Jahnigen S. Copland D. A. Darkow R. Grittner U. Avirame K. (2014). Transcranial direct current stimulation over multiple days improves learning and maintenance of a novel vocabulary. *Cortex* 50 137–147. 10.1016/j.cortex.2013.07.013 23988131

[B39] Michalareas G. Vezoli J. Van Pelt S. Schoffelen J. M. Kennedy H. Fries P. (2016). Alpha-beta and gamma rhythms subserve feedback and feedforward influences among human visual cortical areas. *Neuron* 89 384–397. 10.1016/j.neuron.2015.12.018 26777277 PMC4871751

[B40] Mottaghy F. M. Hungs M. Brugmann M. Sparing R. Boroojerdi B. Foltys H. (1999). Facilitation of picture naming after repetitive transcranial magnetic stimulation. *Neurology* 53 1806–1812. 10.1212/WNL.53.8.1806 10563632

[B41] Mottaghy F. M. Sparing R. Topper R. (2006). Enhancing picture naming with transcranial magnetic stimulation. *Behav. Neurol.* 17 177–186. 10.1155/2006/768413 17148838 PMC5471541

[B42] Naeser M. A. Martin P. I. Theoret H. Kobayashi M. Fregni F. Nicholas M. (2011). TMS suppression of right pars triangularis, but not pars opercularis, improves naming in aphasia. *Brain Lang.* 119 206–213. 10.1016/j.bandl.2011.07.005 21864891 PMC3195843

[B43] Najib U. Bashir S. Edwards D. Rotenberg A. Pascual-Leone A. (2011). Transcranial brain stimulation: clinical applications and future directions. *Neurosurg. Clin. N. Am.* 22 233–251 ix 10.1016/j.nec.2011.01.002 21435574 PMC3547606

[B44] New B. Pallier C. Brysbaert M. Ferrand L. (2004). Lexique 2: a new French lexical database. *Behav. Res. Methods Instrum. Comput.* 36 516–524. 10.3758/BF03195598 15641440

[B45] Newman M. E. (2004). Analysis of weighted networks. *Phys. Rev. E* 70 056131 10.1103/PhysRevE.70.056131 15600716

[B46] Nicolo P. Ptak R. Guggisberg A. G. (2015). Variability of behavioural responses to transcranial magnetic stimulation: origins and predictors. *Neuropsychologia* 74 137–144. 10.1016/j.neuropsychologia.2015.01.033 25619851

[B47] Noh N. A. Fuggetta G. Manganotti P. Fiaschi A. (2012). Long lasting modulation of cortical oscillations after continuous theta burst transcranial magnetic stimulation. *PLoS ONE* 7:e35080 10.1371/journal.pone.0035080 PMC3319628 22496893

[B48] Nolte G. Bai O. Wheaton L. Mari Z. Vorbach S. Hallett M. (2004). Identifying true brain interaction from EEG data using the imaginary part of coherency. *Clin. Neurophysiol.* 115 2292–2307. 10.1016/j.clinph.2004.04.029 15351371

[B49] Nyffeler T. Wurtz P. Pflugshaupt T. Von Wartburg R. Luthi M. Hess C. W. (2006). One-Hertz transcranial magnetic stimulation over the frontal eye field induces lasting inhibition of saccade triggering. *Neuroreport* 17 273–275. 10.1097/01.wnr.0000199468.39659.bf 16462596

[B50] Oldfield R. C. (1971). The assessment and analysis of handedness: the Edinburgh inventory. *Neuropsychologia* 9 97–113. 10.1016/0028-3932(71)90067-4 5146491

[B51] Pedersen P. M. Vinter K. Olsen T. S. (2004). Aphasia after stroke: type, severity and prognosis. The Copenhagen aphasia study. *Cerebrovasc. Dis.* 17 35–43. 10.1159/000073896 14530636

[B52] Pfurtscheller G. (1992). Event-related synchronization (ERS): an electrophysiological correlate of cortical areas at rest. *Electroencephalogr. Clin. Neurophysiol.* 83 62–69. 10.1016/0013-4694(92)90133-3 1376667

[B53] Pfurtscheller G. Andrew C. (1999). Event-Related changes of band power and coherence: methodology and interpretation. *J. Clin. Neurophysiol.* 16 512–519. 10.1097/00004691-199911000-00003 10600019

[B54] Piai V. Roelofs A. Maris E. (2014). Oscillatory brain responses in spoken word production reflect lexical frequency and sentential constraint. *Neuropsychologia* 53 146–156. 10.1016/j.neuropsychologia.2013.11.014 24291513

[B55] Piai V. Roelofs A. Rommers J. Maris E. (2015). Beta oscillations reflect memory and motor aspects of spoken word production. *Hum. Brain Mapp.* 36 2767–2780. 10.1002/hbm.22806 25872756 PMC6869587

[B56] Protopapas A. (2007). CheckVocal: a program to facilitate checking the accuracy and response time of vocal responses from DMDX. *Behav. Res. Methods* 39 859–862. 10.3758/BF03192979 18183901

[B57] Raboyeau G. De Boissezon X. Marie N. Balduyck S. Puel M. Bezy C. (2008). Right hemisphere activation in recovery from aphasia: lesion effect or function recruitment? *Neurology* 70 290–298. 10.1212/01.wnl.0000287115.85956.87 18209203

[B58] Reis J. Robertson E. M. Krakauer J. W. Rothwell J. Marshall L. Gerloff C. (2008). Consensus: can transcranial direct current stimulation and transcranial magnetic stimulation enhance motor learning and memory formation? *Brain Stimul.* 1 363–369. 10.1016/j.brs.2008.08.001 PMC2621080 20633394

[B59] Restle J. Murakami T. Ziemann U. (2012). Facilitation of speech repetition accuracy by theta burst stimulation of the left posterior inferior frontal gyrus. *Neuropsychologia* 50 2026–2031. 10.1016/j.neuropsychologia.2012.05.001 22580417

[B60] Rizk S. Ptak R. Nyffeler T. Schnider A. Guggisberg A. G. (2013). Network mechanisms of responsiveness to continuous theta-burst stimulation. *Eur. J. Neurosci.* 38 3230–3238. 10.1111/ejn.12334 23941616

[B61] Sekihara K. Nagarajan S. S. Poeppel D. Marantz A. (2004). Performance of an MEG adaptive-beamformer source reconstruction technique in the presence of additive low-rank interference. *IEEE Trans. Biomed. Eng.* 51 90–99. 10.1109/TBME.2003.820329 14723498

[B62] Sekihara K. Owen J. P. Trisno S. Nagarajan S. S. (2011). Removal of spurious coherence in MEG source-space coherence analysis. *IEEE Trans. Biomed. Eng.* 58 3121–3129. 10.1109/TBME.2011.2162514 21824842 PMC4096348

[B63] Silvanto J. Muggleton N. Walsh V. (2008). State-dependency in brain stimulation studies of perception and cognition. *Trends Cogn. Sci.* 12 447–454. 10.1016/j.tics.2008.09.004 18951833

[B64] Silvanto J. Pascual-Leone A. (2008). State-dependency of transcranial magnetic stimulation. *Brain Topogr.* 21 1–10. 10.1007/s10548-008-0067-0 18791818 PMC3049188

[B65] Spinelli L. Andino S. G. Lantz G. Seeck M. Michel C. M. (2000). Electromagnetic inverse solutions in anatomically constrained spherical head models. *Brain Topogr.* 13 115–125. 10.1023/A:1026607118642 11154101

[B66] Stam C. J. van Straaten E. C. (2012). The organization of physiological brain networks. *Clin. Neurophysiol.* 123 1067–1087. 10.1016/j.clinph.2012.01.011 22356937

[B67] Tanaka S. Sandrini M. Cohen L. G. (2011). Modulation of motor learning and memory formation by non-invasive cortical stimulation of the primary motor cortex. *Neuropsychol. Rehabil.* 21 650–675. 10.1080/09602011.2011.605589 21942897

[B68] Thiel A. Hartmann A. Rubi-Fessen I. Anglade C. Kracht L. Weiduschat N. (2013). Effects of noninvasive brain stimulation on language networks and recovery in early poststroke aphasia. *Stroke* 44 2240–2246. 10.1161/STROKEAHA.111.000574 23813984

[B69] Thiel A. Herholz K. Von Stockhausen H. M. Van Leyen-Pilgram K. Pietrzyk U. Kessler J. (1998). Localization of language-related cortex with 15O-labeled water PET in patients with gliomas. *Neuroimage* 7 284–295. 10.1006/nimg.1998.0334 9626669

[B70] Topper R. Mottaghy F. M. Brugmann M. Noth J. Huber W. (1998). Facilitation of picture naming by focal transcranial magnetic stimulation of Wernicke’s area. *Exp. Brain Res.* 121 371–378. 10.1007/s002210050471 9746143

[B71] Tzourio-Mazoyer N. Landeau B. Papathanassiou D. Crivello F. Etard O. Delcroix N. (2002). Automated anatomical labeling of activations in SPM using a macroscopic anatomical parcellation of the MNI MRI single-subject brain. *Neuroimage* 15 273–289. 10.1006/nimg.2001.0978 11771995

[B72] Vallence A. M. Goldsworthy M. R. Hodyl N. A. Semmler J. G. Pitcher J. B. Ridding M. C. (2015). Inter- and intra-subject variability of motor cortex plasticity following continuous theta-burst stimulation. *Neuroscience* 304 266–278. 10.1016/j.neuroscience.2015.07.043 26208843

[B73] Veniero D. Brignani D. Thut G. Miniussi C. (2011). Alpha-generation as basic response-signature to transcranial magnetic stimulation (TMS) targeting the human resting motor cortex: a TMS/EEG co-registration study. *Psychophysiology* 48 1381–1389. 10.1111/j.1469-8986.2011.01218.x 21542853

[B74] Vernet M. Bashir S. Yoo W. K. Perez J. M. Najib U. Pascual-Leone A. (2013). Insights on the neural basis of motor plasticity induced by theta burst stimulation from TMS-EEG. *Eur. J. Neurosci.* 37 598–606. 10.1111/ejn.12069 23190020 PMC4191847

[B75] Wessel M. J. Zimerman M. Timmermann J. E. Heise K. F. Gerloff C. Hummel F. C. (2016). Enhancing consolidation of a new temporal motor skill by cerebellar noninvasive stimulation. *Cereb. Cortex* 26 1660–1667. 25604611 10.1093/cercor/bhu335

[B76] Winhuisen L. Thiel A. Schumacher B. Kessler J. Rudolf J. Haupt W. F. (2005). Role of the contralateral inferior frontal gyrus in recovery of language function in poststroke aphasia: a combined repetitive transcranial magnetic stimulation and positron emission tomography study. *Stroke* 36 1759–1763. 10.1161/01.STR.0000174487.81126.ef 16020770

[B77] Wong P. C. Perrachione T. K. Parrish T. B. (2007). Neural characteristics of successful and less successful speech and word learning in adults. *Hum. Brain Mapp.* 28 995–1006. 10.1002/hbm.20330 17133399 PMC6871292

