# Supplementary material for: Neurobiological Correlates of Inhibition of the Right Broca Homolog during New-Word Learning
Source: Front Hum Neurosci. 2016 Jul 28;10:371. doi: 10.3389/fnhum.2016.00371 (PMC4963391; doi:10.3389/fnhum.2016.00371)
Supplement: Supplementary file 1 [file Data_Sheet_1.DOCX]

Supplemental Material to

**Inhibition of right Broca homologue does not improve verbal learning**

Pierre Nicolo^1^, Raphaël Fargier^3^, Marina Laganaro^3^ and Adrian G. Guggisberg^1,2^

*^1^Division of Neurorehabilitation, Department of Clinical Neurosciences, Geneva University Hospitals, Geneva, Switzerland*

^2^Laboratory of Cognitive Neurorehabilitation, Department of Clinical Neurosciences, Medical School, University of Geneva, Geneva, Switzerland

^3^Faculty of Psychology and Educational Sciences, University of Geneva, Switzerland.

**Supplemental Materials and Methods**

Stimuli and Subjects

Example of Experimental Stimuli

Two lists of 50 words corresponding to rare objects were used in the experiment. Below are some examples of black and white line drawings used for assessment and colored or photo pictures used for training.

| 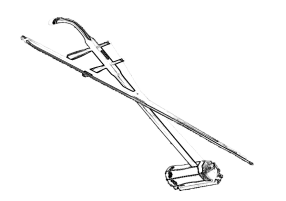 | 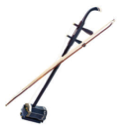 | **Ehru** |
| --- | --- | --- |
| 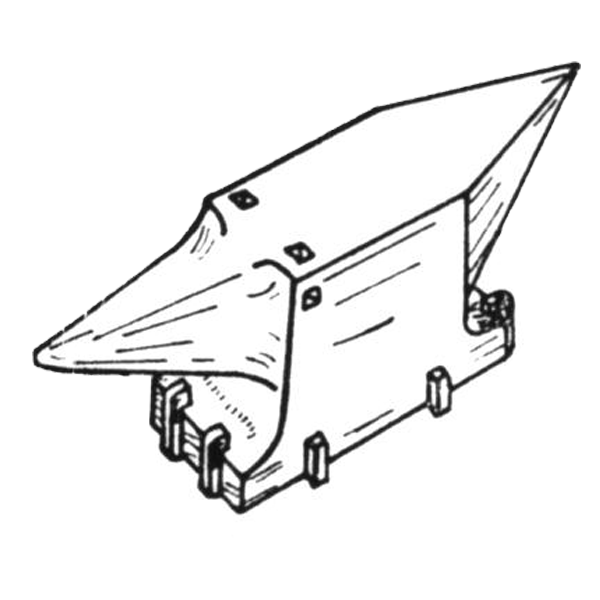 | 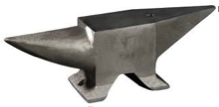 | **Enclume** |
| 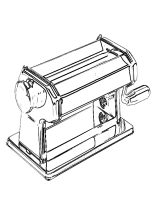 | 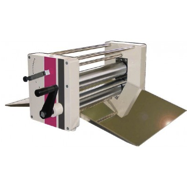 | **Laminoir** |
| 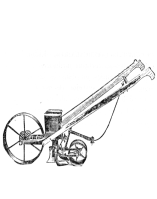 | 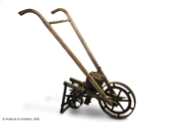 | **Semoir** |
| 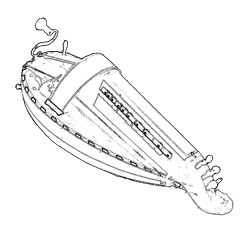 | 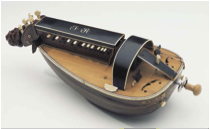 | **Vielle** |

**Supplementary Tables**

**Table S1**. Participant characteristics.

| **Subjects** | **Genre** | **Age** | **L1** | **L2** | **Visual Acuteness** | **Laterality** | **Educational Level** |
| --- | --- | --- | --- | --- | --- | --- | --- |
| 102 | W | 23 | French |  | Corrected | SD10 | Master |
| 103 | W | 22 | French |  | Corrected | SD10 | Bachelor |
| 104 | W | 21 | French |  | Good | SD8 | Bachelor |
| 105 | M | 25 | French |  | Corrected | SD8 | Bachelor |
| 106 | W | 22 | French |  | Good | SD8 | Bachelor |
| 107 | W | 21 | French |  | Good | SD9 | Bachelor |
| 108 | W | 21 | French |  | Good | SD9 | Bachelor |
| 109 | W | 21 | French | Portuguese | Corrected | SD9 | Bachelor |
| 110 | W | 19 | French | English | Good | SD9 | Bachelor |
| 111 | M | 22 | French |  | Good | SD8 | Bachelor |
| 112 | W | 28 | French | German, Italian | Corrected | SD10 | Master |
| 113 | M | 32 | French |  | Good | SD10 | Doctorate |
| 114 | W | 31 | French |  | Corrected | SD9 | Bachelor |
| 115 | W | 25 | French |  | Good | SD9 | Bachelor |
| 116 | W | 35 | French |  | Good | SD9 | Master |
| 117 | M | 32 | French |  | Good | SD9 | Bachelor |

**Table S-2**. Psycholinguistic properties (mean) of stimuli in the two lists (from the French database Lexique ([New *et al.*, 2004](#_ENREF_1))). Frequencies are per million words.

|  | Oral Lemma Frequency | Written Lemma frequency | Oral Lexeme Frequency | Written Lexeme Frequency | Length in phonemes | Length in syllables | Phonological Neighbors |
| --- | --- | --- | --- | --- | --- | --- | --- |
| List A | 0.46 | 1.61 | 0.36 | 1.07 | 5.72 | 2.22 | 4.34 |
| List B | 0.25 | 0.95 | 0.22 | 0.66 | 5.64 | 2.16 | 4.42 |
|  |  |  |  |  |  |  |  |
| *(unpaired t-test p)* | *0.39* | *0.37* | *0.42* | *0.32* | *0.80* | *0.95* | *0.70* |

**Reference**

New, B., Pallier, C., Brysbaert, M. & Ferrand, L. (2004) Lexique 2: a new French lexical database. *Behav Res Methods Instrum Comput*, **36**, 516-524.
